# Beyond X-Rays: Unveiling the Future of Dental Diagnosis with Dental Magnetic Resonance Imaging

**DOI:** 10.3390/diagnostics15091153

**Published:** 2025-05-01

**Authors:** Anusha Vaddi, Pranav Parasher, Sonam Khurana

**Affiliations:** 1 Department of Oral Diagnostic Sciences, School of Dentistry, Virginia Commonwealth University, 520 N 12th Street, Richmond, VA 23298, USA; 2 Section of Oral and Maxillofacial Radiology, Division of Oral and Maxillofacial Diagnostic Sciences, UConn School of Dental Medicine, UConn Health, 263 Farmington Avenue, Farmington, CT 06030, USA; parasher@uchc.edu; 3 Department of Oral and Maxillofacial Pathology, Radiology & Medicine, College of Dentistry, New York University, 345 East 24th Street, New York, NY 10010, USA; sk10542@nyu.edu

**Keywords:** dental MRI, dental magnetic resonance imaging, Ultrashort Echo Time (UTE), Zero Echo Time (ZTE), Sweep Imaging with Fourier Transformation (SWIFT), intraoral coils, head and neck coils, surface coils

## Abstract

Diagnostic imaging is fundamental in dentistry for disease detection, treatment planning, and outcome assessment. Traditional radiographic methods, such as periapical and panoramic radiographs, along with cone beam computed tomography (CBCT), utilize ionizing radiation and primarily focus on visualizing bony structures. Magnetic resonance imaging (MRI) is emerging as a non-ionizing alternative that offers superior soft tissue contrast. However, standard MRI sequences face challenges visualizing mineralized tissues due to their short transverse relaxation times (T2), which results in rapid signal decay. Recent advancements exploring short T2 sequences, including Ultrashort Echo Time (UTE), Zero Echo Time (ZTE), and Sweep Imaging with Fourier Transformation (SWIFT), allow direct visualization of dental hard tissues. UTE captures signals from short T2 tissues using rapid pulse sequences, while ZTE employs encoding gradients before radiofrequency pulses to reduce signal loss. SWIFT enables near-simultaneous excitation and acquisition, improving ultrashort T2 detection. Additionally, customized intraoral and extraoral surface coils enhance the image resolution and signal-to-noise ratio (SNR), increasing MRI’s relevance in dentistry. Research highlights the potential of these short T2 sequences for early caries detection, pulp vitality assessment, and diagnosing jaw osseous pathology. While high-field MRI (3 T–7 T) improves resolution and increases susceptibility artifacts, low-field systems with specialized coils and short sequences offer promising alternatives. Despite obstacles such as cost and hardware constraints, ongoing studies refine protocols to enhance clinical applicability. Incorporating MRI in dentistry promises a safer, more comprehensive imaging methodology, potentially transforming diagnostics. This review emphasizes three types of short T2 sequences that have potential applications in the maxillofacial region.

## 1. Introduction

Diagnostic imaging plays a crucial role in dentistry by providing essential anatomical information that complements clinical examinations in the detection and assessment of oral and maxillofacial diseases. Imaging technology influences all aspects of oral healthcare, ranging from monitoring disease progression to treatment planning and assessment of therapeutic outcomes [1]. Despite their significance, dental practices predominantly rely on ionizing radiation-based imaging modalities, which pose cumulative radiation exposure risks [1,2]. Periapical, bitewing, panoramic radiographs, and cone beam computed tomography (CBCT) are the most commonly used imaging methods in dentistry. However, these imaging techniques often fail to visualize soft tissues [3]. Both 2D and 3D imaging approaches may not detect early inflammatory changes, which typically precede noticeable bone loss [4]. For radiographic identification of these alterations using conventional radiographs, significant mineral loss (30–50%) is usually necessary to observe periapical and periodontal lesions [5]. Although advanced 3D imaging techniques, particularly CBCT, provide an enhanced understanding of anatomical structures and pathology, they still involve ionizing radiation, even when protocols aim to minimize doses [6]. The intricate anatomy of the dentomaxillofacial region poses difficulties for current imaging techniques due to the combination of different types of hard and soft tissues and air and fluid-filled spaces. This region encompasses critical anatomical structures, such as the maxilla, mandible (including the neurovascular canals), nose, paranasal sinuses, and oral cavity, including teeth [7]. Magnetic Resonance Imaging (MRI) is gaining recognition for imaging the head and neck and evaluating the dentoalveolar complex. Traditionally, it has been used in head and neck imaging to examine the temporomandibular joint (TMJ), salivary glands, and soft tissue abnormalities. MRI offers benefits over conventional radiography, including the absence of ionizing radiation and better soft tissue differentiation [8]. The absence of ionizing radiation in MRI makes it an ideal method for continuous research and repeated scans in children and young adults, particularly at risk from the cumulative effects of ionizing radiation [9,10]. Although there are some contraindications for MRI, especially at higher magnetic field strengths, dental materials and orthodontic devices are typically not prohibited. However, these items may create artifacts that can potentially affect the quality of the images produced [11]. Recent MRI advancements that reduce the time between signal excitation and acquisition now enable osseous imaging within minutes, which previously took hours. These techniques hold great promise for dental MRI. Ex vivo Ultrashort Echo Time (UTE) and Zero Echo Time (ZTE) MRI outperform conventional MRI and CBCT in both resolution and detail. Notably, these methods can differentiate multiple components within the solid structure of the tooth, an area that previously yielded no detectable signal [3]. This review outlines the advancements in dental MRI, highlighting the potential of UTE, ZTE, and Sweep Imaging with Fourier Transformation (SWIFT) sequences in overcoming the limitations of conventional imaging techniques. By discussing the role of intraoral coils and the expanding applications of MRI in dentistry, this article emphasizes the evolving landscape of diagnostic imaging. As these innovations continue to refine image quality and clinical utility, dental MRI stands poised to transform oral healthcare by providing a non-ionizing, high-resolution alternative for both hard and soft tissue assessment.

## 2. Challenges and Limitations of Conventional MRI in Dental Imaging

MRI is a powerful, non-invasive imaging modality for diagnosing and monitoring soft tissue conditions without exposing patients to ionizing radiation. It works by detecting signals from hydrogen nuclei in water molecules, commonly referred to as the “water signal”. This signal is generated when a radiofrequency (RF) pulse excites nuclear spins, causing them to resonate within a strong static magnetic field. However, conventional MRI struggles to capture detailed images of teeth due to their high mineral content. Dentin is composed of roughly 50% minerals by volume, while enamel consists of about 90%, with the remainder made up of water and proteins. This composition limits the visibility of dental structures in standard MRI scans [12]. Additionally, the restricted molecular motion of water within these highly mineralized structures leads to rapid signal decay after RF excitation, known as free induction decay (FID). This decay is characterized by the transverse relaxation time (T2), which averages approximately 200 μs for dentin [13] and 60 μs for enamel [14]. These short decay times fall well below the duration required for conventional MRI pulse sequences to complete spatial encoding using pulsed magnetic field gradients, which typically require over 1 ms. Consequently, the signal from mineralized dental tissues fades before MRI signal digitization, making these structures appear dark or black in MRI images. As a result, traditional MRI applications in dentistry have been restricted to imaging the pulp, attached periodontal membrane, and surrounding soft tissues or have depended on indirect visualization of enamel and dentin using MRI-visible contrast agents [15,16,17].

## 3. Advances in Short T2 MRI Sequences for Dental Hard Tissue Visualization

Recent advancements in pulse sequence technology have enabled the visualization of highly mineralized structures such as enamel, dentin, and bone, which were previously difficult to image using conventional MRI. These techniques, commonly referred to as short T2 sequences, are designed to capture the rapidly decaying T2 signal characteristic of hard tissues. Among the most notable short T2 sequences are Ultrashort Echo Time (UTE), Zero Echo Time (ZTE), and Sweep Imaging with Fourier Transformation (SWIFT), all of which have demonstrated promising results in dental imaging [18].

### 3.1. Ultrashort Echo Time (UTE)

UTE imaging encompasses specialized MRI sequences optimized for tissues with ultra-short T2 relaxation times (under 10 ms) and sometimes as brief as 0.01–0.1 ms. The conventional 2D UTE typically employs a half-RF pulse excitation with a slice-selective gradient, followed by a second acquisition with an opposite gradient to achieve full slice excitation. Data acquisition starts immediately with gradient activation, capturing radial k-space trajectories (typically 128–512 steps) that are reconstructed using a 2D Fourier Transform into a high-resolution image matrix (e.g., 512 × 512) [19,20,21,22,23].

In contrast, 3D UTE utilizes a non-selective RF pulse and samples k-space volumetrically using radial, spiral, or spherical trajectories, which enables isotropic spatial resolution and is particularly well-suited for imaging entire structures composed of short T2 species, such as cortical bone, tendons, and dental tissues [24]. Compared to 2D UTE, 3D UTE offers volumetric imaging, improved signal-to-noise ratio (SNR), and greater uniformity [25]. However, these advantages come with technical challenges: the rapid gradient switching and short sampling durations required for UTE can induce eddy currents and image artifacts—A common issue in all non-Cartesian MRI methods [26,27]. Solutions such as pre-emphasis correction, imaging-based gradient measurements (IGM), and gradient impulse response functions (GIRF) are employed to mitigate these distortions [28]. Additionally, advanced techniques like ramped hybrid encoding (RHE) have been developed, in which gradients are ramped up following an initial low-gradient excitation to minimize slice selectivity and reduce sampling duration [29]. RHE-UTE has demonstrated improved spatial resolution and fewer artifacts, and when combined with dynamic single-point imaging (SPI), it further enhances k-space sampling fidelity and image quality [30].

### 3.2. Zero Echo Time (ZTE)

In ZTE imaging, the encoding gradient is activated before the RF pulse, theoretically resulting in a zero echo time (TE). This technique employs a short hard-pulse excitation with a small flip angle while the gradients in all three spatial directions are gradually adjusted. Compared to UTE, ZTE sequences impose greater limitations on flip angles and readout bandwidths. However, the gradual gradient adjustments contribute to significantly reduced acoustic noise and mitigate eddy current effects, making ZTE a highly stable imaging method. Data acquisition follows a 3D radial center-out approach, with k-space reconstruction performed through gridding and Fourier Transform (FT), similar to UTE imaging. In practice, a short delay (δ) occurs after RF excitation due to the time required for transmit/receive switching. To compensate for this data gap, an oversampling technique is applied using a linear algebra-based reconstruction scheme. While effective, large-scale oversampling increases data volume, necessitating substantial computational memory for processing [31,32].

To better understand the evolution of ZTE, it is important to consider its foundational concepts and historical development. ZTE imaging emerged from earlier techniques such as BLAST (Back-projection Low Angle ShoT) and RUFIS (Rotating Ultra-Fast Imaging Sequence), which pioneered the use of low flip angle RF pulses and direct acquisition of free induction decays (FIDs) rather than traditional echoes. RUFIS, in particular, utilized a constant magnetic field gradient whose direction was rotated between acquisitions, enabling image reconstruction via back-projection with minimal demands on gradient performance. This approach offered significant advantages, including insensitivity to motion, flow, and diffusion, making it especially suitable for imaging dynamic processes and turbulent flow. RUFIS also achieved faster acquisition rates than other methods available at the time, such as Echo Planar Imaging (EPI), Dual Flip Angle Ultra Short Echo Time Imaging with Saturation (DUFIS), Optimized Ultra-Fast Imaging Sequence (OUFIS), and Bipolar UTE with Residual Saddle Term (BURST), while reducing artifacts related to stimulated echoes. These innovations laid the groundwork for modern ZTE techniques, which continue to leverage echo-free acquisition strategies and gradient-rotation principles to achieve high-speed, motion-robust imaging with reduced system constraints and expanded clinical applications [33,34].

To enhance k-space filling, an alternative method known as Pointwise Encoding Time Reduction with Radial Acquisition (PETRA) has been introduced [35]. This approach integrates radial mapping with Cartesian single-point acquisition, enabling accurate filling of the central k-space region. PETRA allows for improved SNR by capturing more signals from short T2 tissues before significant decay occurs. However, this method requires the imaged object to fit within the primary lobe of the sinc-shaped excitation profile. To address potential artifacts caused by inhomogeneous excitation, a correction algorithm using a quadratic phase-modulated RF pulse has been developed, allowing for extended RF pulses with lower peak power, making PETRA more suitable for clinical MRI systems. PETRA-based imaging has demonstrated superior performance compared to conventional ZTE techniques and has been successfully implemented in 1.5 T and 3 T MRI systems for hard tissue imaging [36,37].

Despite its advantages, ZTE imaging faces technical challenges, including the need for rapid RF switching and robust hardware capable of handling large data volumes. Recent advancements have led to the development of custom imaging consoles equipped with high-speed spectrometers, pulse generators, and real-time data storage solutions. These enhancements have facilitated reductions in RF excitation pulses and transmit/receive switching times. However, concerns remain regarding the large excitation bandwidth, specific absorption rate (SAR), and associated imaging artifacts. Further refinements, such as using amplitude- and frequency-modulated pulses for broader RF excitation and improved suppression of long T2 signals, have significantly advanced ZTE imaging for clinical applications [38,39].

### 3.3. Sweep Imaging with Fourier Transformation (SWIFT)

The SWIFT technique, introduced by Idiyatullin et al., integrates key elements of three fundamental Nuclear Magnetic Resonance (NMR) approaches: continuous wave (CW), pulsed, and stochastic NMR. It employs a swept RF excitation similar to CW NMR but at a faster rate, acquires signals over time like pulsed NMR, and utilizes correlation-based extraction methods characteristic of stochastic NMR. This combination enables an MRI sequence capable of nearly simultaneous excitation and acquisition, making it particularly effective for detecting ultrashort T2 components.

In SWIFT imaging, RF pulses are applied in sequences with a designated duration (Tp) in the millisecond range. Each pulse is segmented into multiple parts, during which the RF signal remains active for a specific time interval (τp) following a brief delay. Signal acquisition occurs at τa after each segment, allowing for the collection of frequency-encoded projections. These projections are subsequently reconstructed using a 3D back-projection or gridding algorithm, followed by image formation through a cross-correlation method. However, the time required for transitions between pulse segments imposes constraints on signal-to-noise ratio (SNR) and image resolution [40,41].

Several refinements of the SWIFT sequence have been developed to help mitigate hardware constraints, lower specific absorption rate (SAR), and improve acquisition efficiency, enhancing the overall performance of SWIFT imaging. The advancements include continuous SWIFT (cSWIFT), multiple excitation bands (MB-SWIFT), and gradient-modulated SWIFT (GM-SWIFT) [42,43,44].

## 4. Dental MRI Coils: Advancements and Challenges in Imaging Quality

Dental MRI offers high-contrast imaging of soft tissues and specialized imaging of hard tissues such as teeth and bones. However, achieving high spatial resolution remains challenging due to the low signal-to-noise ratio (SNR) inherent in small voxel volumes. The performance of dental MRI is critically dependent on both radiofrequency (RF) coils for signal detection and gradient coils for spatial encoding [45].

### 4.1. Radiofrequency Coils

1. Body Coils:

Clinical MRI scanners are typically equipped with large RF coils within the bore, known as body coils, designed to energize the entire body. These body coils transmit radio waves regardless of the anatomy being imaged. Anatomy-specific receiver or surface coils record the RF signal emitted from the patient.

2. Surface Coils:

Surface coils are placed adjacent to the anatomy being imaged, enhancing the detection of the RF signal. In dental MRI, anatomy-specific coils, such as extraoral or intraoral coils, improve resolution by focusing on a smaller Field of View (FOV). These coils allow for higher-resolution imaging, crucial for detecting small anatomical changes in dental structures, such as the teeth, periodontium, and alveolar bone [46].

### 4.2. Innovative Coil Designs for Dental MRI Radiofrequency Intraoral Coils

Cable-bound intraoral coils, placed between teeth, have demonstrated resolutions of 300 μm^3^ with a 12 cm^3^ FOV in 4.5 min. However, these designs struggle to capture critical structures like the periapical region or alveolar bone [47].

1. Wireless Inductively-Coupled Coils:

These coils offer localized sensitivity with resolutions ranging from 350 to 800 μm^3^. Their wireless design improves patient comfort and reduces setup complexity, making them a promising option for dental MRI [48].

2. Multi-Channel RF Arrays:

A 7-channel RF coil array with flexible “wings” has been developed to enhance imaging performance while prioritizing patient comfort. This design allows for angular adjustments for optimal placement near target areas and supports parallel imaging, which enables faster acquisition times [49].

3. SWIFT Coils:

SWIFT technology utilizes specialized intraoral coils to simultaneously image both hard and soft tissues. Invivo studies have demonstrated the feasibility of using a one-sided shielded single-loop intraoral coil (40 mm diameter) placed between the cheek and teeth, secured with a 2 mm thick Teflon bite fork for stable positioning. The coil’s close proximity to the region of interest enhances SNR, improving resolution while maintaining clinically feasible scan times. The reduced gradient demands of SWIFT also make it a promising option for low-noise, high-resolution dental imaging [47].

4. Transverse Loop Coils (tLoop and mtLoop)

The transverse loop coil (tLoop) introduced an innovative design for dental MRI, with the coil plane orthogonal to B_0_, enabling full dental arch imaging while minimizing signals from adjacent tissues like the cheeks and tongue [50]. Despite its advantages, the tLoop faced limitations such as steep sensitivity gradients, signal voids at the incisors due to feed port gaps, and discomfort caused by its rigid geometry. The modified transverse loop coil (mtLoop) was developed to address these issues with significant improvements. These include overlapping feed port conductors to eliminate signal voids, a bent posterior section for enhanced comfort, and a parallel plate capacitor to reduce eddy currents by 10%. Operating in receive-only mode, the mtLoop achieves an 11× SNR improvement at incisors and 2.5× at molar roots, enabling isotropic resolutions of 250 μm^3^ within a 2-min scan time. These advancements make the mtLoop a promising solution for high-resolution, patient-friendly dental imaging [51].

5. Coupled Stack-Up Volume Coils:

Coupled stack-up volume coils enhance RF field efficiency for low-field MRI systems by ensuring strong coupling between coil elements. This improves both transmit/receive efficiency and field homogeneity, making it effective for dental imaging in lower-field systems [52].

### 4.3. Challenges in Dental MRI Radiofrequency Coils

1. Low SNR and Resolution:

Current clinical MRI systems, despite having advanced gradient hardware capable of 0.2 mm resolution, still struggle to match the spatial resolution of Multi-detector Computed Tomography (MDCT) or CBCT. SNR is directly affected by voxel size, making it challenging to balance resolution with imaging time

2. Design Limitations:

Standard surface coils, commonly used for dental MRI, face challenges such as handling difficulties and reduced patient comfort. Conventional head-and-neck coils often have a low filling factor for dental imaging, reducing sensitivity.

3. Material and Miniaturization Challenges:

Coils must be biocompatible, lightweight, and ergonomically designed for intraoral or facial use. These requirements complicate manufacturing, especially as miniaturization is necessary for high-quality dental imaging.

4. FOV Limitations:

In conventional MRI setups—particularly when using standard head or neck coils—the FOV often encompasses the entire head, which limits spatial resolution and makes it challenging to detect dental structures. However, recent surface and intraoral coil design advancements have enabled more focused imaging with smaller, anatomy-specific FOVs. These targeted approaches improve resolution by isolating the region of interest, although they may still face limitations in capturing broader anatomical contexts or when precise positioning is not possible [50].

### 4.4. Gradient Coils

Gradient coils embedded within the MRI magnet housing generate spatially varying magnetic fields along the *x*-, *y*-, and *z*-axes, essential for spatial encoding and image reconstruction [32]. In dental MRI, achieving high-resolution imaging presents unique challenges, particularly due to the technical demands placed on gradient coil performance. Rapid gradient switching, necessary for some advanced pulse sequences, can generate significant acoustic noise that may affect patient comfort. Each sequence (UTE, ZTE, and SWIFT) imposes distinct requirements on the gradient system. UTE sequences require highly efficient gradients with fast ramp times to capture signals from tissues with very short T2 relaxation times. In contrast, ZTE and SWIFT sequences operate with gradients that are continuously on or rapidly switched, necessitating hardware capable of supporting high-duty cycles and wide bandwidths. However, they place less emphasis on ramp speed [32,40]. Among these, SWIFT is particularly well-suited for dental applications due to its reduced gradient switching demands, lower acoustic noise, and capability for high-resolution imaging of both hard and soft tissues [40].

## 5. Clinical Applications of Short T2 Sequences in Dentistry

In dentistry, MRI has traditionally been employed to diagnose internal derangements related to the TMJ. MR imaging can reveal the disc shape, position, and direction of displacement, as well as any signs of inflammation, such as effusion, and to some extent, indicate degenerative joint disease. Additionally, MRI was utilized to assess infectious or inflammatory conditions in the maxillofacial region, examine soft tissue pathologies of the salivary glands, maxillary sinuses, and masticatory muscle abnormalities, and identify vascular anomalies and lesions [53]. As previously discussed, short sequences like UTE, ZTE, and SWIFT have greatly enhanced the visualization of mineralized dental tissues, including enamel, dentin, and bone. These sequences open new opportunities for dental MRI, enabling a thorough evaluation of hard tissues and soft tissue structures. The following section explores the potential uses of these sequences in dentistry, emphasizing their importance in diagnosing and monitoring a wide range of dental conditions.

## 6. Imaging of Teeth

The use of MRI in dentistry is steadily increasing, but imaging dental tissues remains challenging due to their short relaxation times. The first UTE MR image of teeth was captured in 2003 using the Fat-suppressed UTE (FUTE) method on a 1.5 T system (Gatehouse & Bydder, 2003) [54]. Later, whole-body 3 T MRI scans were conducted on human subjects with a total scan time of 10 min. UTE sequences with a TE of 50 μs provided detailed visualization of dental structures, including enamel. Furthermore, 3D-UTE imaging allowed for high-resolution tooth anatomy visualization and helped estimate relative T2* values, which have been linked to the detection of dental caries [55].

**Figure 1:**
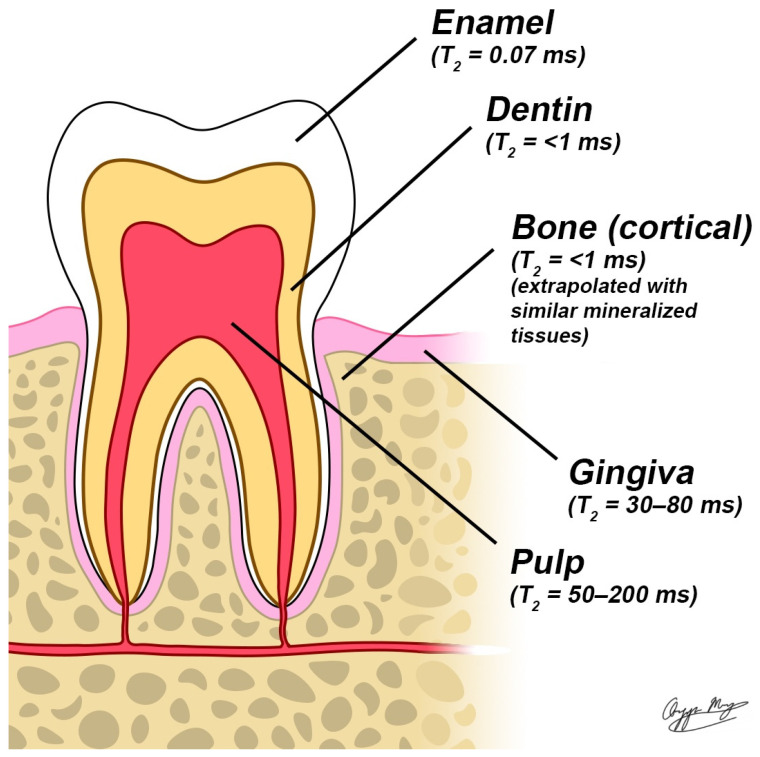
A schematic of human molar anatomy highlight senamel, dentin, pulp, gingiva, and bone. T2 relaxation times for each structure are mentioned using a 1.5 T MRI system, providing valuable insights into their structural properties.

The SWIFT images provide a detailed visualization of dental anatomy, including enamel, dentin, and pulpal structures. Due to its lower water content and shorter T2 relaxation time, enamel exhibits a less intense signal than dentin. The observed signal intensities in the pulp, dentin, and enamel follow an approximate ratio of 100:35:10. This pattern is consistent with their respective water content levels, roughly 100:20:8 [47]. Because MRI signal strength is strongly influenced by the amount of water in tissues, structures with higher water content, such as the pulp, appear much brighter than those with lower water content, like enamel. This correlation reinforces the reliability of MRI in reflecting differences in dental tissue composition. These SWIFT images lack significant T2 weighting, unlike Gradient Echo (GRE) images, allowing for clear visualization of all dental tissues [54] (Figure 1).

### 6.1. Detection of Dental Caries

In the early stages of dental caries, the decayed area experiences acid buildup, leading to the demineralization of the tooth, which increases tissue porosity and potentially allows saliva to seep in. These factors raise local proton density and prolong the T2 spins of protons. The resulting high-intensity signal from water, combined with the absence of signal from adjacent mineralized tissues, creates a contrast that helps distinguish caries from the surrounding tooth structure. However, this signal can only be detected using short TE MR sequences. The random dephasing caused by residual minerals limits the visualization of caries in conventional spin echo images. In more advanced lesions, an increase in T2 values and minimal random dephasing, caused by a notable reduction in mineral content, enhances the visualization of advanced lesions in spin-echo images [55]. In addition, UTE sequences generated less severe metal artifacts than a conventional spin echo. The 3D UTE MRI demonstrated comparable sensitivity to both intermediate and advanced lesions, significantly outperforming X-ray and conventional spin-echo MRI methods for early-stage lesions [56].

### 6.2. Dental Restorative Materials

Using UTE-MRI at 3 T and 9.4 T, researchers have demonstrated the ability to differentiate dental hard tissues and restorative materials based on their unique T1 and T2* relaxation properties and signal intensities. In the 3 T study, dentin exhibited a T1 of 545 ms—significantly longer than restorative materials like Harvard Cement (30 ms) and Provicol QM (166 ms)—due to its higher water and organic content. T2* values for dentin (478 μs) were comparable to materials like Rebilda DC (337 μs), though restorative materials showed greater variability due to differences in composition [57]. In contrast, the 9.4 T UTE-MRI study found that dentin appeared with moderate signal intensity, while gutta-percha showed no signal (signal void), and AH Plus sealer exhibited a slightly higher signal due to its greater hydrogen content and longer T2* relaxation times. These distinct T1 and T2* characteristics enable UTE-MRI to distinguish dentin from filling materials effectively, providing high-resolution imaging that can assess root canal anatomy and material interfaces and detect potential degradation or recurrent caries—something conventional MRI cannot achieve [58].

### 6.3. Dental Pulp and Vitality Status of Tooth

Dental MRI is gaining recognition as a non-invasive and radiation-free tool for assessing pulp vitality and perfusion, especially when standard diagnostic methods like thermal or electric tests provide inconclusive results. Due to similar signal patterns, traditional T_1_ and T_2_ MRI sequences often fall short in differentiating between vital and non-vital pulps. However, contrast-enhanced T_1_-weighted imaging has shown potential in this area. This technique allows for the assessment of pulpal perfusion by tracking changes in signal intensity after contrast administration, with research reporting marked differences in enhancement between vital (82.7%) and non-vital (17.3%) teeth (*p* = 0.003). In regenerative endodontic treatments, newly formed pulp tissue has been found to exhibit MRI signal characteristics that align with cold test outcomes (r = −0.63, *p* < 0.05) [59,60]. Additionally, 3 T MRI has proven effective in visualizing blood flow restoration in pediatric dental trauma, potentially preventing unnecessary endodontic therapy [61].

Advanced MRI sequences such as ZTE, UTE, and SWIFT have significantly enhanced the ability to visualize the internal structure and condition of the dental pulp. Notably, 3D ZTE MRI conducted at ultra-high magnetic field strengths (e.g., 9.4 Tesla) has shown promise in distinguishing between intact and damaged pulp tissue in ex vivo models, with contrast variations corresponding to areas of pulpal degeneration, as demonstrated in equine studies [3]. Although these imaging methods do not directly assess vascular perfusion, they are highly effective in revealing morphological changes such as calcification, resorption, or structural compromise of the pulp chamber [3].

### 6.4. Periapical Inflammatory Disease

MRI has proven highly effective in differentiating periapical cysts from granulomas and monitoring their healing process after treatment. In their 2023 study, Wamasing et al. [62] identified several key MRI features for distinguishing these lesions, including lesion size (with cysts typically measuring 15.9 mm or larger), margin clarity (well-defined in cysts and ill-defined in granulomas), and peripheral rim thickness (thin in cysts and thick in granulomas). These findings are consistent with the work of Juerchott et al. in 2018 [63], who outlined six important MRI criteria, such as the texture of the peripheral rim (homogeneous in cysts versus heterogeneous in granulomas) and the signal characteristics of the lesion center on T2-weighted fat-saturated sequences (uniform in cysts, variable in granulomas). For post-treatment monitoring, MRI is valuable for assessing healing, with key indicators including reduced T2 hyperintensity (signifying edema resolution) and normalized bone marrow T1 signal intensity (reflecting osseous repair), as reported by Wamasing et al. [62]. Additionally, persistent contrast enhancement or the presence of recurrent fluid-filled cavities may indicate incomplete healing or recurrence of the lesion [62,63]. However, despite the growing interest in advanced sequences such as SWIFT, UTE, and ZTE MRI, there remains a lack of clinical studies specifically evaluating their role in diagnosing and monitoring periapical lesions.

### 6.5. Periodontal Assessment

A recent technical report demonstrated the potential of a dental-dedicated magnetic resonance imaging (ddMRI) system for non-invasive periodontal assessment. This imaging modality effectively depicted changes in marginal bone levels, offering important information about the progression of periodontal disease and the effectiveness of therapeutic interventions. The ddMRI system also identified advanced periodontal involvement, such as furcation defects marked by bone loss between tooth roots. Beyond anatomical visualization, the ddMRI sequences detected signal changes consistent with fluid accumulation in the periodontal tissues, which may indicate ongoing inflammation. These observations highlight the supplementary role of ddMRI in clinical evaluations, particularly in identifying structural alterations and signs of active inflammatory processes in periodontal conditions [49].

### 6.6. Cracks and Fracture Detection in the Teeth

Cracks in teeth often present a diagnostic challenge. While CBCT enhances the detection of these cracks, assessing their proximity to dental restorations remains difficult due to artifacts. In teeth with crowns, these artifacts can obscure both the crown and the crack in CBCT images. An ex vivo study involving extracted teeth compared CBCT with SWIFT and GRE protocols, utilizing intraoral coils and MicroCT as a reference. The authors found that SWIFT MRI could detect cracks as narrow as 20 μm, which is more than ten times smaller than the image voxel size. They also noted that MR images are less influenced by metal restorations like amalgam and gold, resulting in fewer artifacts than in CBCT. The authors attributed this advantage to the presence of water in the crack, which serves as a negative contrast, and the differences in transverse relaxation times compared to motion-restricted mineralized tissues like dentin. Since the T2 relaxation time of water is in the submillisecond range, cracks are not visible in GRE images obtained under similar conditions [64]. The presence of artifacts in filled teeth makes it difficult to detect cracks in CBCT images. In vitro studies have demonstrated that UTE sequences can successfully identify dentin cracks in endodontically treated teeth [58]. Similar to cracks, artifacts due to restorative materials pose a challenge in diagnosing root fractures and vertical root fractures (VRF). In vitro studies assessing root fractures and VRFs using MRI have shown comparable specificity and sensitivity to those obtained with small FOV CBCT [65,66]. Notably, studies utilizing SWIFT MRI sequences have successfully detected VRFs with widths ranging from 26 to 64 µm [67].

### 6.7. Nerve Detection

On CBCT, visualization of the inferior alveolar nerve (IAN) often relies on indirect assessment through the cortical boundaries of the mandibular canal. However, in individuals with conditions such as osteopenia, reduced bone mineral density, or osseous pathology like osteolytic lesions, these cortices may undergo resorption or disruption, compromising the reliability of nerve tracing. In contrast, MRI allows for direct visualization of the IAN and lingual nerve, independent of surrounding bone [68]. High-resolution MRI sequences such as 3D double-echo steady-state (DESS) and short-tau inversion recovery (STIR) offer excellent soft-tissue contrast for imaging neurovascular structures within the mandible. Research indicates that DESS sequences are particularly effective in visualizing the inferior alveolar neurovascular bundle and peripheral branches of the trigeminal nerve, benefiting from high signal-to-noise ratios and minimal susceptibility to dephasing artifacts. Additionally, SPACE-STIR sequences enhance nerve visualization by suppressing fat signals, thereby improving contrast in regions affected by magnetic field variations from dental appliances [69]. In a technical report focused on ddMRI, the authors noted that the images also successfully demonstrated the lingual nerve in addition to a clear depiction of the inferior alveolar nerve (IAN) [49].

### 6.8. Alveolar Bone Evaluation for Implant Treatment Planning

Accurate measurements of the implant site are crucial for effective implant planning. CBCT is the preferred method for obtaining bone measurements before implant placement. The ZTE sequences were assessed for their capability to evaluate bone height, width, and area at the implant site. Research conducted on human cadavers showed that bone height measurements were nearly equivalent to those obtained from CBCT. However, ZTE MRI slightly underestimated bone width and area at the implant site [70]. In another similar study, alveolar bone height and width measurements in ZTE MR images were comparable to those from CBCT [71].

### 6.9. Jaw Bone Pathology

While evaluating both odontogenic and non-odontogenic jaw lesions, short echo time (TE) sequences, such as Zero Echo Time (ZTE) MRI, can provide diagnostic information comparable to that of multidetector computed tomography (MDCT) in assessing lesion margins, internal architecture, and effects on adjacent anatomical structures. When ZTE sequences are combined with conventional MRI sequences—including T1-weighted imaging for evaluating marrow fat content, T2-weighted imaging and STIR (Short Tau Inversion Recovery) for detecting fluid and edema, diffusion-weighted imaging (DWI) for assessing tissue cellularity, and contrast-enhanced T1-weighted imaging for evaluating vascularity—comprehensive tissue characterization can be achieved. This combined approach can reveal critical features such as mineralization within the internal matrix, which is suggestive of calcified cysts or tumors, and fluid–fluid levels within lesions, a characteristic finding in entities like aneurysmal bone cysts. Additionally, the inherently reduced imaging time of ZTE sequences enhances their utility in neonates and pediatric patients, where motion artifacts can be challenging, facilitating the detection of bone abnormalities and vascular malformations [72].

Similarly, in evaluating medication-related osteonecrosis of the jaw (MRONJ), UTE sequences have been explored for their diagnostic utility compared with CBCT. The qualitative assessment analyzed characteristics such as osteolysis, periosteal thickening, and osteosclerosis. A significant correlation was observed between the two modalities across both qualitative and quantitative parameters. However, the air in the vestibule close to the bone appeared similarly dark to the bone in UTE MR sequences, which posed a challenge. Adequate instructions and rinsing the mouth before the scan might minimize these artifacts [73].

### 6.10. Temporomandibular Joint (TMJ)

MRI is the preferred method for assessing internal derangements of the TMJ. MRI provides valuable insights into disc position, morphological changes of the disc, joint effusion, and some signs of degenerative joint disease. Additionally, it can reveal alterations in surrounding soft tissues and muscles. Over the years, various MRI techniques have been developed to explore biochemical and structural changes near the joint that occur before visible morphological changes.

The application of short sequences, such as UTE, has improved the visualization of structures with short T2, including the fibrocartilaginous disc and the fibrocartilage lining the articular surfaces of the joint. Studies have indicated that the mean UTE T2 in symptomatic individuals is higher than in asymptomatic individuals. Further research is necessary to clarify the potential role of these sequences in evaluating TMJ pathology [74]. ZTE sequences have demonstrated encouraging outcomes, compared to CBCT, in assessing degenerative changes in the joint, including flattening, sclerosis, osteophyte formation, and remodeling alterations in the fossa. However, sufficient data is lacking to confirm these results [75].

### 6.11. Potential Uses in Oral Cancer

Sequences such as ZTE and UTE are FDA-approved for clinical use and can be utilized at various magnetic field strengths. Using surface coils positioned directly over the area of interest is advisable. MRI, with its superior contrast resolution, effectively highlights tumor margins and surrounding tissues, including muscle, fascia, and perineural fat. Typically, MDCT imaging is conducted alongside MRI to assess bone involvement. Integrating ZTE sequences eliminates the necessity for extra MDCT scans, facilitating accurate evaluation and tumor staging. Another significant benefit of ZTE is its capability to evaluate postoperative complications due to reduced hardware-related artifacts [64]. A study on oral cancer patients assessed the effectiveness of Positron Emission Tomography (PET)/CT and PET/MRI with ZTE-based attenuation correction (ZTE-AC). The ZTE-AC maps accurately defined the jaw bones, and the impact of metal artifacts from dentures was minimal [76].

### 6.12. MR-Based Panoramic Reconstruction

Panoramic imaging is a two-dimensional X-ray technique utilized as a screening tool that comprehensively assesses the dentoalveolar region. A typical digital panoramic image captured with a charge coupled device (CCD) exposes the patient to a radiation dose ranging from 14 to 24 microsieverts [77].

In CBCT, curved panoramic reconstructions, also known as curved planar reformats, can be produced with varying slice thicknesses, attaining as thin as 1 mm or even less, depending on the voxel size and software configurations. However, for clinical panoramic-like images, it is typical to increase the thickness to between 10 and 25 mm to simulate traditional panoramic projections. In contrast, MR-panoramic reconstructions are created using a curved multiplanar reconstruction (MPR) technique. This involves manually defining a curve along the dental arch within the 3D MRI dataset. Thin slices, usually about 0.5 mm, are extracted orthogonally to this curve at small intervals, and these are sequentially combined to produce a panoramic reconstruction. Utilizing this thin-slice method is crucial in MRI, as it helps maintain anatomical detail and reduce soft tissue overlap. Summative reconstructions over thicker slabs, often used in CBCT, are not as effective in MRI because of the low signal intensity from mineralized tissues and the high contrast by soft tissues. Some researchers have approached this issue with specialized imaging techniques such as ‘black bone MRI’ or traditional GRE imaging with short TE. These initiatives focused primarily on bone segmentation. Moreover, they often necessitate extra equipment or multiple imaging sequences [78].

In a pilot study to produce panoramic reformats, researchers employed a 3 T scanner and a 15-channel mandibular coil, experimenting with sequences such as UTE and DESS (Double Echo Steady State) in patients undergoing a third molar extraction. The UTE-based sequences demonstrated superior performance in evaluating spatial relationships and root morphology, with excellent image quality and reduced susceptibility artifacts, making it as the most precise MR representation compared to the conventional panoramic X-ray. Additionally, the DESS MR panoramic image has demonstrated to be effective for neurography, especially for imaging the IAN and the lingual nerve [79].

### 6.13. MR-Based Lateral Cephalometric Reconstruction and Cephalometric Analysis

Lateral cephalograms (LC) are frequently utilized in orthodontics to conduct cephalometric analysis. Various types of analyses rely on identifying specific osseous anatomical landmarks. A standard lateral cephalometric image subjects the patient to a 5–6 microsievert radiation dose. Most patients receiving orthodontic treatment are typically in their growth phase, making them ideal candidates for non-ionizing radiation-based imaging techniques. In the realm of MRI, some researchers have experimented with adjustments to the GRE scan protocol. They reduced the flip angle and decreased the repetition time (TR) and echo time (TE). This imaging technique called “black bone” MRI, enhanced the contrast between bone and soft tissue in MR images [80]. In another study, the authors modified a similar protocol to create 2D midsagittal planes and compared these with traditional LC, T1, and T2 sequences. A limitation of this study is that anatomical information from the paramedian planes was inaccessible. A subsequent effort was undertaken to gather data from median and paramedian slices by incorporating UTE sequences, further minimizing TR and TE. While the MR-generated LC successfully identified various anatomical landmarks, the traditional LC outperformed it. The authors attributed this difference to lower resolution, increased scan noise, and insufficient training in interpreting these novel images [81].

### 6.14. Assessment of Craniocervical Junction

MDCT remains the preferred imaging method for assessing alterations in the craniocervical junction (CCJ). Recent advancements in bone visualization due to the development of specialized sequences have led some studies to explore the efficacy of shorter sequences for evaluating the CCJ. UTE demonstrated a high level of agreement for specific distance measurements compared to MDCT. Given these findings, UTE is suggested as a viable alternative to MDCT [82].

### 6.15. Applications in Forensic Dentistry

The formation of secondary dentin in teeth has been utilized as a parameter for estimating age. One key measurement is the reduction in the size of the pulp cavity observed through imaging. Research demonstrated that UTE sequences at high magnetic field strengths (9.4 T) effectively distinguish between tooth structure and pulp in extracted teeth, achieving an in-plane resolution of 66 μm^3^. Using these tooth-to-pulp volume ratios, calculations can aid in age estimation [83]. In a separate study, the authors compared measurements with CBCT, noting slight variations; specifically, the pulp volume appeared smaller on MRI. They concluded that method-specific reference values are essential for practical age assessment [84].

In most of the aforementioned applications, short sequences demonstrated potential comparable to ionizing radiation-based modalities. However, most studies are based on in vitro, ex vivo, and cadaveric research. Studies involving human subjects typically had small sample sizes. Nevertheless, further research is necessary to validate these findings.

## 7. Discussion

The first NMR image was created by Lauterbur in 1973. Since that time, the field has experienced remarkable progress. While MRI scans of the head and neck can reveal the teeth and dentoalveolar region, MRI systems have not been adopted in routine dental practice due to inadequate visualization of mineralized tissues. Furthermore, several obstacles, such as space constraints in dental operatories, cost, and technological challenges, have hindered its implementation in dentistry. However, advancements in hardware, including the development of light-magnet technology and built-in automation, and the refinement of short T2 sequences have made MRI more accessible to dentistry, similar to X-ray-based imaging.

Numerous efforts have been made to enhance the visualization of dentoalveolar structures. These efforts focus on the use of contrast agents, high field strengths, the development of specialized surface coils, and the enhancement of imaging sequences. A study conducted in 2004 investigated different contrast agents by filling patient’s mouths with biocompatible materials. The authors noted that some of these contrast materials improved the visibility of anatomical structures while reducing susceptibility artifacts. Furthermore, they indicated that the spatial resolution of contrast-enhanced dental MRI is on par with MDCT scans [15].

Magnetic field strength is measured in Tesla (T), with most systems utilized in clinical settings falling between 1.5 and 4 T. Units operating at 3–7 T are classified as high Tesla. Numerous previous studies indicate that 3 T MRI scanners provide enhanced visualizations of dental structures [51,85,86]. The heightened magnetic field strength increases the signal-to-noise ratio (SNR) and enhances image resolution. However, with higher field strengths, there is a decrease in T2 relaxation time and an increase in susceptibility artifacts that may hinder in vivo resolution. New generation scanners now focus on imaging teeth at low field strengths such as 0.55 T, placement of surface coils, and ultra-short echo time sequences [49,87,88]. Recent research by Nixdorf et al. (2025) [89] has shown that a 0.55 T MRI system designed for dental use generated TMJ images that are subjectively comparable in quality to those from a conventional 1.5 T system. In their research, despite variations in coils and software versions, evaluators from different specialties did not consistently note significant differences in image quality or the visibility of anatomical landmarks. These results indicate that, with further optimization, low-field MRI systems could present a viable alternative for TMJ imaging [89].

Traditionally, MRI scans of the gnathic region are conducted using head and neck coils. With their large FOV and limited gradient strength, these coils compromised the image resolution, making them less effective for examining teeth and endodontic structures. In recent years, specialized MRI coils for dentistry have been developed. These include both extraoral surface coils and intraoral coils. These advancements minimize the FOV and have shown encouraging results for imaging teeth and surrounding structures [43,54,90]. Research indicates that using intraoral coil placement enhances image resolution; however, this approach has its drawbacks. It can interfere with anatomical structures and presents challenges in coil placement, often resulting in difficulties capturing distal areas, root apices, and periapical regions [91]. Studies showed significant improvement in SNR with surface coils positioned directly on the face and mandible [92].

As previously discussed, specific types of sequences with short T2 values eliminate the signal void caused by mineralized tissues that have high dipole-dipole interaction. In vitro studies utilizing such sequences demonstrated the ability to accurately evaluate the level of demineralization and identify occlusal and recurrent caries and cracks within the tooth [41].

Advanced MRI sequences such as UTE and ZTE provide innovative alternatives to conventional MDCT and other dental imaging techniques. These sequences enable simultaneous visualization of both soft and hard tissues, allowing clear differentiation of dental structures, including enamel, dentin, cementum, and pulp. At higher magnetic field strengths, such as 7 T, they enhance image contrast and resolution beyond what is achievable with standard MRI and CBCT.

ZTE MRI has shown high accuracy in depicting solid dental structures, closely matching actual tooth surfaces. It effectively distinguishes between healthy and diseased pulp regions. It can visualize materials like calcium phosphate-based pulp capping and glass ionomer restorations, which are typically challenging to detect using conventional MRI.

Unlike traditional UTE sequences, the SWIFT technique utilizes swept RF excitation and simultaneous signal acquisition while maintaining field gradients. This method allows for the imaging of ultrashort T2 components with minimal strain on MR hardware, making it particularly effective for capturing calcified dental tissues. Additionally, SWIFT excels in identifying accessory canals in the apical third of the root, offering superior diagnostic capabilities in endodontics compared to CBCT [93].

In a recent study, the authors utilized a scanner developed specifically for dentistry. The machine operates at a magnetic field strength of 0.55 T and is equipped with a dental surface coil positioned on the face. This setup minimizes the FOV and employs a series of optimized MRI pulse sequences specifically designed for common dental diagnostic procedures. To address diverse requirements in dentistry, certain pulse sequences create 3D volumetric outputs from proton density-weighted sequences designed for effectively visualizing anatomy. In contrast, other sequences yield 2D image slabs customized with T1, T2, or Proton density weightings, depending on the particular diagnostic needs. Preliminary findings from the report indicate a diverse array of applications in orthodontics, including the generation of 2D lateral cephalograms to identify cephalometric points for analysis, panoramic reconstructions akin to CBCT, detection of carious tooth, fractures in crowns and roots, and endodontic evaluations of root canals and periapical tissues. Furthermore, it is valuable in assessing periodontal hard and soft tissues [49].

## 8. Conclusions

This paper outlines the critical developments supporting the integration of MRI into dental diagnostics, marking a shift toward high-resolution, radiation-free imaging. Advances in magnetic field optimization, intraoral and surface coil design, and short T2 sequences (UTE, ZTE, and SWIFT) have significantly improved the ability to visualize both hard and soft tissues with clinically feasible scan times and high diagnostic accuracy. Dental-specific MRI systems now address the anatomical and technical challenges of the dentoalveolar region, enhancing image quality and reducing artifacts. These improvements are expanding MRI’s potential applications in dental diagnostics, particularly in areas requiring simultaneous assessment of hard and soft tissues—where conventional imaging may offer limited information.

The absence of ionizing radiation enables safe, repeat imaging, which is especially advantageous for pediatric patients and individuals requiring longitudinal follow-up. Future work should focus on protocol optimization, artifact reduction, and hardware improvements to support broader clinical implementation. Continued interdisciplinary collaboration will be essential to advance dental MRI from research to routine practice. In conclusion, the integration of advanced MRI technology into dental imaging holds promise for redefining diagnostic approaches, offering a non-invasive, comprehensive alternative that supports improved patient care.

## Abbreviations

The following abbreviations are used in this manuscript:

CBCT	Cone Beam Computed Tomography
MRI	Magnetic Resonance Imaging
UTE	Ultrashort Echo Time
ZTE	Zero Echo Time
SWIFT	Sweep Imaging with Fourier Transformation
TMJ	Temporomandibular Joint
RF	Radiofrequency
FID	Free Induction Decay
SNR	Signal-to-Noise Ratio
IGM	Imaging -Based Gradient Measurements
GIRF	Gradient Impulse Response Function
RHE	Ramped Hybrid Encoding
TE	Echo Time
FT	Fourier Transform
PETRA	Pointwise Encoding Time Reduction with Radial Acquisition
EPI	Echo Planar Imaging
DUFIS	Dual Flip Angle Ultra Short Echo Time Imaging with Saturation
OUFIS	Optimized Ultra- Fast Imaging Sequence
BURST	Bipolar UTE with Residual Saddle Term
BLAST	Back-projection Low Angle ShoT
RUFIS	Rotating Ultra-Fast Imaging Sequence
SAR	Specific Absorption Rate
NMR	Nuclear Magnetic Resonance
PET	Positron Emission Tomography
CW	continuous wave
FOV	Field of View
tLoop	transverse loop coil
mtLoop	modified transverse loop coil
FUTE	Fat-Suppressed UTE
GRE	Gradient Echo Images
IAN	Inferior Alveolar Nerve
DESS	Double Echo Steady State
MPR	Multiplanar Reconstruction
CT	Computed Tomography
MRONJ	Medication-Related Osteonecrosis of Jaws
CCD	Charge-Coupled Device
LC	Lateral cephalogram
TR	Repetition Time
CCJ	Craniocervical Junction
